# First detection of a cervidpoxvirus in Europe—disease occurrence in semi-domesticated Eurasian tundra reindeer (*Rangifer tarandus tarandus*) in Norway and Sweden

**DOI:** 10.1128/spectrum.02296-24

**Published:** 2025-05-22

**Authors:** Ingebjørg H. Nymo, Cathrine Arnason Bøe, Javier Sánchez Romano, Hans Lian, Renate Thorvaldsen, Faisal Suhel, Mette Boye, Øivind Øines, Lars P. Folkow, Hans-Arne Solvang, Tomas Jinnerot, Jonas Johansson Wensman, Rebecca K. Davidson, Torill Mørk, Line Olsen, Morten Tryland, Ulrika Rockström, Bjørn Spilsberg

**Affiliations:** 1 Department of Animal Health, Welfare and Food Safety, Norwegian Veterinary Institute, Tromsø, Norway; 2 Department of Arctic and Marine Biology, UiT — The Arctic University of Norway, Tromsø, Norway; 3 Department of Analysis and Diagnostics, Norwegian Veterinary Institute, Ås, Norway; 4 Institute of Zoology, Zoological Society of London, London, United Kingdom; 5 Department of Medical Biology, UiT — The Arctic University of Norway 198675 , Tromsø, Norway; 6 Department of Microbiology, Swedish Veterinary Agency, Uppsala, Sweden; 7 Department of Animal Biosciences, Swedish University of Agricultural Sciences, Uppsala, Sweden; 8 Department of Forestry and Wildlife Management, University of Inland Norway, Koppang, Norway; 9 Farm & Animal Health, Uppsala, Sweden; University of Pittsburgh, Pittsburgh, Pennsylvania, USA

**Keywords:** Emerging infectious diseases, cytopathic effect, histopathology, *in situ *hybridization, *in vitro*, next generation sequencing, periorbital lesions, PCR, stress, poxvirus

## Abstract

Globally, there are six millions of *Rangifer* spp., including both wild and semi-domesticated populations. Reindeer herding with Eurasian tundra reindeer (*Rangifer tarandus tarandus*) is central to the Fennoscandian Sámi people. Commencing in 2018, a novel disease presenting with periorbital lesions was reported in reindeer in multiple locations in Norway and Sweden, as individual cases and outbreaks. Lesions were also seen on the genitals, nostrils, and ears of affected reindeer, in combination with lethargy. Necropsy findings included lesions with crusts on the eyelids, vulva, and anus, along with moderately swollen lymph nodes, liver, and spleen. Histological examination of the eyelid lesions showed a disrupted epidermis with necrotic squamous epithelium and inflammation. Shotgun sequencing of samples from outbreaks yielded a partial draft genome of a virus related to Deerpox virus W-848-83 (currently known as *Cervidpoxvirus muledeerpox*) in genus *Cervidpoxvirus*. Cervidpoxviruses have previously only been detected in Capreolinae in North America. An in-house qPCR targeting the cervidpoxvirus virion core protein gene was designed and showed the presence of viral-specific DNA in multiple organs, with the highest load in skin lesions. RNAscope *in situ* hybridization detected viral transcripts in the epithelium of the eye lesions. Transmission electron microscopy analysis revealed the presence of intracytoplasmic poxvirus-like particles with characteristic morphology within the lesions. *In vitro* infection in Madin-Darby bovine kidney epithelial cells demonstrated cytopathic effect. It is unknown whether the emergence of this disease in semi-domesticated reindeer in Norway and Sweden is due to ecosystem changes facilitating altered disease dynamics, or if the virus has been recently introduced.

**IMPORTANCE:**

This study documents the first detection of a Cervidpoxvirus in Europe, causing disease in semi-domesticated reindeer in Norway and Sweden. It identifies the causative agent and describes the disease characteristics of this previously unknown condition in reindeer, advancing our understanding of how this virus impacts reindeer health and welfare. Given the cultural and ecological importance of reindeer herding in Fennoscandia, understanding this disease is crucial for safeguarding the livelihoods of Sámi communities. Additionally, the study raises important questions about the role of ecosystem changes, climate-driven insect dynamics, and potential reservoir hosts in facilitating disease outbreaks. These findings underscore the broader implications of environmental change on animal health and highlight the need for continued research to mitigate risks posed by emerging infectious diseases (EIDs).

## INTRODUCTION

Globally, there are approximately six million *Rangifer* spp., including both wild and semi-domesticated populations (1). Eurasian tundra reindeer (*Rangifer tarandus tarandus*) exist as wild and semi-domesticated in Fennoscandia, and reindeer herding is a cultural cornerstone for the indigenous Sámi people (2). Norway has around 215,000 semi-domesticated reindeer (3) and 25,000 wild reindeer (1), while Sweden has approximately 240,000 semi-domesticated reindeer (4). Due to historical herding practices and shared grazing rights, some reindeer herds move between Norway and Sweden (5).

Reindeer herding traditionally involves pastoralism, whereby the animals graze extensive rangelands and move between seasonal pastures. This system is heavily dependent on pasture availability and quality (2); however, anthropogenic activities fragment pasturelands (6, 7) and predators cause stress, both making pastures less available (8). Climate change exacerbates these challenges (6). The health of semi-domesticated reindeer in Norway (9) and Sweden (10) is generally regarded as good, but the combined effects of these stressors may alter this (11).

In the past decades, feeding and corralling semi-domesticated reindeer during winter has become increasingly common (12), as a response to pasture loss, both due to ice-locked pastures and anthropogenic disturbances (6), and also predator threats (8). This is often associated with stress, increased animal-to-animal contact and more challenging hygienic conditions (12), which can all contribute to the occurrence and spread of infectious diseases (13). Traditional herding, including long migrations, mixing of herds, as well as shared transport and handling facilities, can also increase disease spread (14).

The *Poxviridae* family consists of large, enveloped, brick-shaped, double-stranded DNA viruses. The *Chordopoxvirinae* subfamily consists of 18 genera infecting vertebrates (15). Contagious ecthyma in reindeer, caused by *Parapoxvirus orf* (13) or *Parapoxvirus pseudocowpox* (16), occurs in Fennoscandia (13, 16). Poxvirus-like infections, different from those caused by parapoxviruses, were first described in 1976 in reindeer at Toronto Zoo, Canada (17). The causative agent was identified as a cervidpoxvirus in 2005 (18) and officially recognized as a new virus within the *Cervidpoxvirus* genus in the *Chordopoxvirinae* subfamily in 2006 (15). Cervidpoxviruses have also been detected in other North American Capreolinae (19–26).

Two publications from Toronto Zoo have described cervidpoxvirus infection in reindeer. These were characterized by periorbital crusting, crusting on the muzzle, vulva, and legs as well as varying degrees of keratoconjunctivitis (17, 24). Woodland caribou (*Rangifer tarandus caribou*) kept at the same zoo exhibited similar signs (24), but no further diagnostic efforts were reported.

Clinical signs of cervidpoxvirus infection in other Capreolinae vary. Lesions on skin and mucus membranes have included ulcers, plaques, or crust-covered areas around the eyes, lips, muzzle, nostrils, tongue, oral cavity, and on the extremities, abdomen, and neck. Eye lesions have included corneal ulceration and conjunctivitis. Peritonitis, with transmural ulcers in the rumen, and bronchopneumonia and ulceration of the oesophagus with fibrinopurulent inflammation have been reported. Clinical presentation ranges from mild depression, reduced appetite, and mild, transient fever to mortalities (17–26).

This study presents the first detection of a partial draft genome of a virus from the genus *Cervidpoxvirus* in Europe, specifically from semi-domesticated reindeer in Norway and Sweden exhibiting clinical signs of disease. It provides a description of the clinical manifestations and pathological findings, including histopathology and RNAscope *in situ* hybridization. Furthermore, it presents the detection of viral genetic material in various organs through qPCR and the outcomes of *in vitro* infection studies.

## MATERIALS AND METHODS

### Samples

In October 2022, a Eurasian tundra reindeer (No-22-80-P92, female, 3 years old) was sampled. The animal was euthanized by the injection of an overdose of medetomidin (1 mg/kg) and ketamine (4 mg/kg) (27), and then the fully anesthetized animal was bled by cutting *vena jugularis*, in compliance with the Norwegian Animal Welfare Act (28).

Reindeer No-22-80-P92 was 1 of 16 reindeer at a research animal facility (approval number 089) at UiT—the Arctic University of Norway (UiT), in Troms County, Norway, out of which 11 were affected by a disease outbreak. The reindeer at the research animal facility are housed in 2,000–7,000 m^2^ outdoor pens with three-meter-tall wire fences and unlimited water access. They are fed with pelleted concentrate feed (FK Reinfôr BAS, Felleskjøpet, Norway), lichens and tree branches, as well as having access to natural pasture in the pens. Contact between reindeer in different pens was possible, both directly through the fence of adjacent pens and indirectly through shared equipment. There was no known contact with any domestic animals or other cervids, but wild birds, rodents, and insects had access to the facilities and the shared environment. In 2022, the facility experienced unusually high rodent and insect activity (pers. obs. H. Lian). Human traffic is restricted to authorized personnel only. The facility mainly breeds its own calves; however, a small number of animals were acquired from local Sámi reindeer herds during 2010–2022. Experimental infections are not conducted at these premises, and the animals were not part of any experimental work when the outbreak occurred.

In October 2023, three Eurasian tundra reindeer (Sw-23-80-P207-16, -19, and -25) with periorbital lesions were sampled at an abattoir in Sweden. These came from a traditional Sámi herd in Jämtland County, Sweden, where multiple animals showed periorbital lesions in autumn 2021, 2022, and 2023.

Samples from No-22-80-P92 and Sw-23-80-P207-16 were examined with histology, shotgun sequencing, RNAscope *in situ* hybridization, qPCR, and *in vitro* studies. Samples from reindeer Sw-23-80-P207-16, -19, and -25 were utilized for a next-generation sequencing pilot experiment.

### Necropsy and histopathology

Reindeer No-22-80-P92 underwent necropsy at The Norwegian Veterinary Institute in Tromsø, Norway. Tissue samples were frozen at −20°C for downstream analyses. Eyelids with lesions and lymph nodes were fixed in 4% formalin and embedded in paraffin wax, sectioned and stained, and examined with hematoxylin and eosin according to standard histological techniques for routine histological examination (29).

### Transmission electron microscopy

For transmission electron microscopy (TEM) analysis, frozen tissue samples from reindeer Sw-23-80-P207-16 (periorbital lesion) and No-22-80-P92 (crusty perianal lesion) were initially fixed in McDowell Trump’s fixative (4% formaldehyde and 1% glutaraldehyde in a phosphate buffer at pH 7.2). The samples were then post-fixed in 1% osmium tetroxide solution in water. Following this, they underwent dehydration through a graded ethanol series (30%–100%) and were embedded in epon resin for ultramicrotomy. Ultrathin sections of 70 nm were cut using a Leica EM UC6 ultramicrotome (Leica Microsystems, Austria) with a Diatome diamond knife (Diatome, Switzerland) and stained with 1% uranyl acetate and Reynolds lead citrate and then examined using a Jeol JEM 1010 transmission electron microscope (Jeol USA, USA) connected to a Morada camera system (Olympus, Germany).

### DNA isolation

All DNA extractions were performed according to the manufacturer’s protocol. DNA was isolated from pooled swab samples of three reindeer from Sweden with periorbital lesions from the same herd and slaughter day: Sw-23-80-P207-16, -19, and -25 with the QIAmp DNA Mini Kit (Qiagen, Germany).

DNA was isolated from buffy coat from reindeer No-22-80-P92 with GenElute-E Single Spin Blood DNA kits (Sigma-Aldrich, USA).

Tissue samples from reindeer No-22-80-P92 were weighed, and subsamples of 15–20 mg were placed in 2.0 mL DNA LoBind Tubes (Sigma-Aldrich) for DNA isolation with a GenElute-E Single Spin Tissue DNA kit (Sigma-Aldrich).

DNA was isolated from 20 µL of each batch of cell culture supernatant diluted in 180 µL PBS (QIAamp DNA Mini Kit, QIAGEN).

DNA quality and quantity were determined using the MySpec (VWR, USA) spectrophotometer and dsDNA Qubit BR Kit (Thermo Fisher Scientific, USA). DNA samples were stored at –70°C until further analysis.

### Next-generation sequencing pilot experiment

Approximately 200 ng DNA, obtained from a pooled sample from reindeer Sw-23-80-P207-16, -19, and -25 (periorbital swab samples), was prepared for Illumina sequencing using the Illumina DNA Prep (Illumina, USA) kit. Shotgun sequencing was performed on a MiSeq using v3 chemistry (Illumina) obtaining 2 × 300 base pair reads.

### Bioinformatic and genomic analysis

An initial analysis of the pilot data was performed with the INSaFlu_TELEVIR (www.insaflu.insa.pt/) virus detection pipeline (30, 31), which identified Deerpox virus (DPV) W-848-83 (currently known as *Cervidpoxvirus muledeerpox*) as a close relative. Base quality in the reads was then recorded with fastQC version 0.11.9 (32). Illumina adapter sequences were removed, and low-quality bases were trimmed with Trimmomatic version 0.39 in paired-end mode (33). The last base was removed, and thereafter the 3′ adapters, followed by removal of low-quality bases. The trimmed reads were mapped to the white-tailed deer poxvirus genome (MF966153.1) with Bowtie2 version 2.5.1, and a partial draft genome was generated with iVAR version 1.4.2 (34).

### RNAScope *in situ* hybridization

Samples from periorbital lesions were formalin-fixed (4%) and paraffin-embedded prior to sectioning (3–5 µm) and mounting on polysine Adhesion Microscope slides (Epredia, USA). The RNAscope probe (V-DPV-C1) was custom-designed by ACDBio (USA), based on the partial draft genome of the cervidpoxvirus isolated from reindeer. The probe consisted of 20 antisense oligo pairs targeting the mRNA transcripts encoding serpin-like protein and ankyrin repeat protein.

RNAscope *in situ* hybridization assays were assessed using RNAscope 2.5 high-definition red assay (ACDBio) according to the manufacturer’s recommendations, including hematoxylin counterstaining. A probe targeting the *dihydrodipicolinate reductase* gene from the *Bacillus subtilis* strain SMY served as a negative control. Whole-slide images were obtained using a Nano Zoomer S210 scanner (Hamamatsu Photonics, Japan).

### Polymerase chain reaction

**Fig 1:**
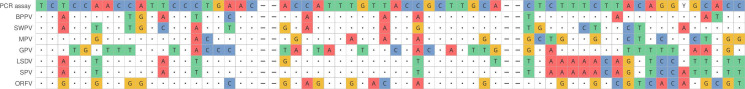
*In silico* specificity for the qPCR detecting cervidpoxvirus in reindeer. The sequences of primers and probe were designed with Primer3Pluss (35) using the partial draft genome as template and aligned to relevant poxviruses in the forward direction. The sequences of the oligonucleotides were separated by two gap symbols. A dot denotes a match, and a base denotes a mismatch. BPPV is Brazilian porcupinepox virus 1 (GCA_031199045.1), SWPV is Swinepox virus isolate 17077-99 (GCF_000839965.1), MPV is Monkeypox virus isolate MPV/A10 (PP265934.1), GPV is Goatpox virus isolate NingX-GY/2011/china (MG458415.1), LSDV is Lumpy skin disease virus isolate N5 (PP405094.1), SPV is Sheeppox virus isolate GanS-JC/2013/China MG458403.1, and ORFV is Orf virus isolate HRE (GCA_030252515.1).

A qPCR method was designed with Primer3Pluss (35) using the partial draft genome as template. Specificity was assessed with PrimerBLAST (36) and manual alignments, and the assay was plotted with ggmsa from Bioconductor version 3.18 and R version 4.3.3 (Fig. 1). The qPCR was designed to target the virion core protein gene in the *Cervidpoxvirus* genus; forward primer reVir-F 5′-TCTCCAACCATTCCCTGAAC-3′, reverse primer reVir-R 5′-GGTGCRCCTGTAAGAAAGAG-3′, and probe reVir_pr 5′-FAM-ACCATTTGTTACCGCTTGCA-BHQ1-3′. The qPCR was performed with Brilliant III Ultra-Fast QPCR Master Mix (Agilent, USA) on an Aria MX instrument (Agilent) with cycling conditions; 95°C, 3 min, and 45× (95°C, 5 s and 60°C, 10 s). A gradient (55–65°C) was performed and showed that 60°C was an acceptable annealing temperature (results not shown). Amplification efficiency was found to be 107.6% and linearity *R*^2^ = 0.995 by analyzing a dilution curve over 5 logs (Fig. S1).

### *In vitro* infection of Madin-Darby bovine kidney cells

Madin-Darby bovine kidney epithelial cells (MDBK; ATCC CCL22) were maintained in Eagle’s minimum essential medium (EMEM; Sigma-Aldrich) supplemented with 5% horse serum (LGC Standards, UK) and 0.01% gentamicin, 0.1% penicillin/streptomycin (P/S), and 0.1% amphotericin B (culture medium) at 37°C with 5% CO_2_.

Approximately 100 mg of crusty lesion from the perianal area of reindeer No-22-80-P92 and the eyelid of reindeer Sw-23-80-P207-16 were homogenized in 1 mL culture medium with a MagNA Lyser instrument (Roche Diagnostics, Switzerland) and MagNA lyser green beads tubes (Roche Diagnostics). Tissues were homogenized twice at 6,000 rpm for 20 s, with a 90 s break between the two steps. The samples were placed on ice immediately after. Supernatants were collected after centrifugation at 2,500 *g* for 15 min at 4°C and stored at −70°C until further use. After retrieval from the freezer, and before inoculation in the cell cultures, supernatants were centrifuged at 2,500 *g* for 15 min at 4°C. Subsequently, 200 µL was inoculated in triplicate wells of a 12-well plate containing monolayers of MDBK cells at approximately 80% confluence and supplemented with 300 µL culture medium, giving a total volume of 500 µL in each well. Culture plates were incubated at 37°C for 1 h for viral adsorption and washed twice with PBS before adding 1,000 µL culture medium to each well. The control wells were inoculated with 200 µL culture medium instead of supernatant, and the procedure above was repeated. Once the cells contained 1,000 µL fresh culture medium, the plates were incubated with 5% CO_2_ at 37°C for 5 days. On day 5 post inoculation (p.i.), culture plates were subjected to three freeze-thaw cycles and centrifugation at 2,500 *g* for 15 min at 4°C, to separate supernatant and cellular debris. A second passage cell culture challenge was performed with 200 µL of the first passage being used as the inoculum and incubated under the same conditions as the first passage.

## RESULTS

### History

In winter 2018, reindeer herders from northern Västerbotten county and southern Norrbotten county in Sweden reported finding single reindeer with periorbital lesions. Since then, similar observations have been made annually in these areas during early winter. When talking to reindeer herders, they shared that single animals with similar lesions had occurred on rare occasions prior to 2018. In the fall of 2021, the first outbreak of periorbital lesions in reindeer from Sweden was reported further south, in Jämtland and Dalarna counties, with a large proportion of the calves observed with periorbital lesions. This happened again during fall 2022, with some spread northward in Jämtland. Once again, the lesions were predominantly observed in calves. In the fall of 2023, much fewer calves with lesions around the eyes were noted as compared to the previous year; however, closer examination during slaughter revealed that ≈30% (*n* ≈ 450) of the male calves had genital lesions (pers. obs. U. Rockström and I. H. Nymo).

### Overview of the disease outbreak in Norway

**Fig 2:**
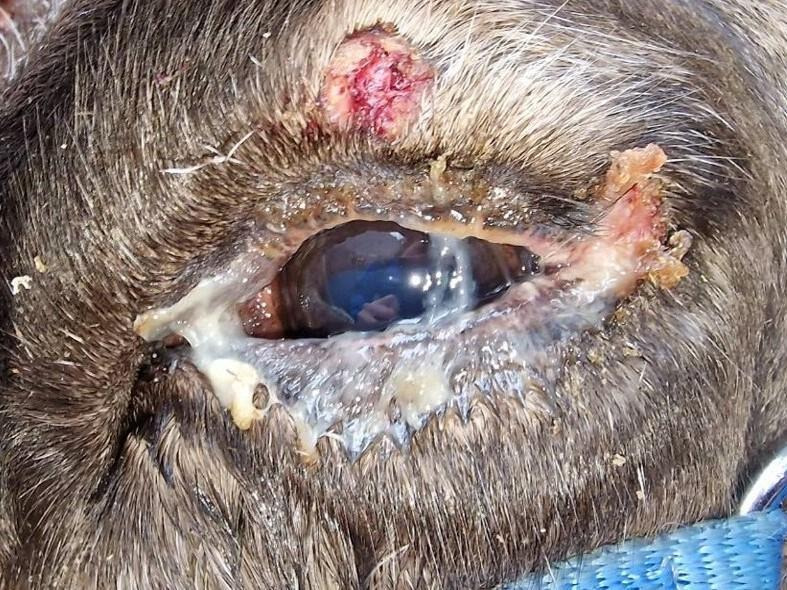
Eye lesions in a semi-domesticated Eurasian tundra reindeer (*Rangifer t. tarandus*) infected with cervidpoxvirus. Ulcer on the lateral canthus of the eye and the middle of the upper eyelid in reindeer 1/21 infected with cervidpoxvirus. Photo: Renate Thorvaldsen, UiT—The Arctic University of Norway.

The disease outbreak in the Norwegian research animal facility included 11 out of the 16 animals in the herd (69%). The outbreak started on 20 September 2022 when a 1-year-old castrated male (animal ID: 1/21) showed increased lacrimation from the left eye. This progressed to ulcers at the lateral canthus and on the middle of the upper eyelid (Fig. 2). On day 2 post symptom (p.s.), crusts started forming, as well as hyperemia and pus in the ulcers, and subcutaneous swelling around the eye. Purulent conjunctivitis was seen in the right eye 3 days p.s. On day 11 p.s., circular, red lesions were seen in the nostrils which progressed to crusts by day 32 p.s. On the same day, crusts were detected in both ears (internal pinnae) and around the preputium. The signs then slowly regressed although increased lacrimation was seen from both eyes until day 89 p.s. Four days after the first clinical signs in the index case (1/21), another 1-year-old castrated male (animal ID: 2/21), kept in the same pen, showed lacrimation and pus in the right eye. Reindeer 2/21 subsequently exhibited a similar disease progression to that observed in 1/21.

A month later, clinical signs were observed in nine additional reindeer in the same herd: two male calves, two female calves, and five adult females. There is some uncertainty regarding the exact timing of the beginning of the disease for each individual, so timings for these are described in broader terms. The first observed clinical signs were periorbital ulcers, with crusting within 1–4 days p.s. Crusts also appeared on the vagina or preputium, as well as hyperemia in the nostrils during the first week. Crusts in the ears appeared by the third week. Subsequently, all signs slowly diminished, and the animals were considered fully healed after ≈ 90 days. The adult females were in good condition when the outbreak started, weighing 104–139 kg, and they lost on average 17 kg (standard deviation: 2.3, range: 86–123 kg) prior to recovery. All animals with clinical signs exhibited various degrees of lethargy during the first weeks. All adult females gave birth to a healthy calf the following spring.

### Treatment and other investigations performed during the outbreak in Norway

The periorbital ulcers were carefully washed 1–2 times/day with sterile physiological saline solution and treated with topical chloramphenicol 10 mg/g (Chloramphenicol, Takeda Pharmaceutical Company, Japan), fusidic acid 10 mg/g (Isathal vet., Dechra Veterinary Products, UK), or cloxacillin 166.6 mg/g (Vetoscon vet., Zoetis, USA) eye ointment at the recommended interval for each. In addition, reindeer 1/21 received cefovecin 1 mL/10 kg s.c. (Convenia vet. 80 mg/mL, Zoetis) twice, while the remaining animals received procaine benzylpenicillin 20 mg/kg i.m. or s.c. (Streptocillin vet., Boehringer Ingelheim, Germany) daily until they showed no signs of secondary infections. The animals also received ketoprofen 3 mg/kg i.m. (Comforion vet. 100 mg/mL, Orion Corporation, Finland, or Romefen vet. 100 mg/mL, Ceva Santé Animale, France) daily for as long as they showed signs of discomfort. Acyclovir 30 mg/g (Xorox, AGEPHA Pharma, France) eye ointment was tried for a restricted period, but had no effect.

Reindeer involved in the outbreak in Norway were investigated for Cervid herpesvirus 2 (CvHV2) DNA and antibodies (37, 38), antibodies against gammaherpesvirus (malignant catarrhal fever group; MCFV) (39, 40), and parapoxvirus DNA (41). All results were negative.

### Gross pathology and histopathology

**Fig 3:**
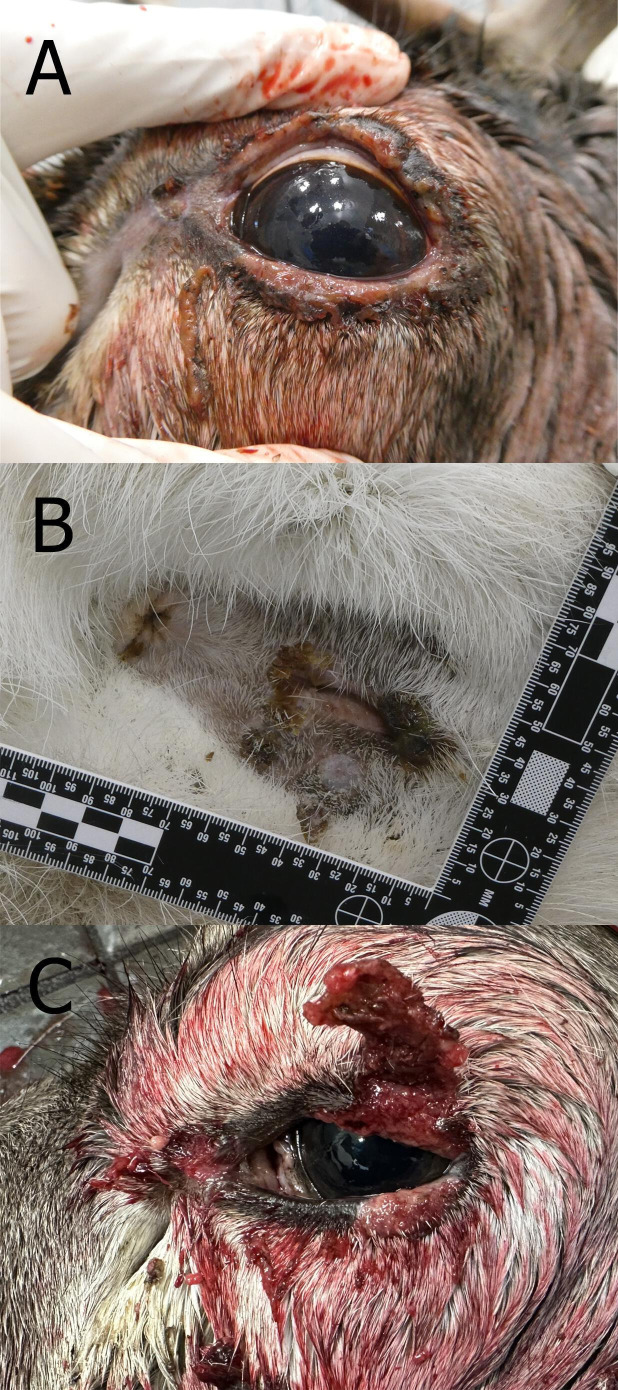
Eye and genital lesions in semi-domesticated Eurasian tundra reindeer (*Rangifer t. tarandus*) infected with cervidpoxvirus. Periorbital lesions around the left eye (**A**) and yellow crusts on the vulva and around the anal opening (**B**) of reindeer No-22-80-P92 from Norway infected with cervidpoxvirus. The animal had yellow crusts around the eye, and the mucocutaneous junction seemed swollen. Similar lesions were found on the upper eyelid (**C**) in a slaughtered reindeer from Sweden (Sw-23-80-P270-16) infected with cervidpoxvirus. Photos: Torill Mørk and Ingebjørg H. Nymo, The Norwegian Veterinary Institute.

Reindeer No-22-80-P92 weighed 93 kg, was in good condition, and had minimal autolytic changes. There were multiple minor lesions, loss of pigmentation, and crusts on the margin of the left upper eyelid and at the medial canthus (Fig. 3A). The crusts were dry, gray-yellow and were easily dislodged. There was no dermal hyperemia underneath the crusts. The whole margin of the upper right eyelid was covered with crusts. There were similar crusts at the mucocutaneous junction around the vulva and anus (Fig. 3B). The prescapular and parotid lymph nodes were moderately enlarged. The liver and spleen were moderately swollen.

The left eye of reindeer Sw-23-80-P207-16 had a large 10 mm diameter crust on the margin of the upper eyelid close to the lateral canthus that partly covered the eye and was easily dislodged. The crust was dry and gray to light red. Underneath the crust was a hyperemic ulcer, and the eyelid was moderately swollen. On the opposing lower eyelid, there was a moderately protruding 10 mm long ulcer along the mucocutaneous junction, with a grayish-white color. In the medial canthus, there was a 5 mm in diameter large ulcer (Fig. 3C).

**Fig 4:**
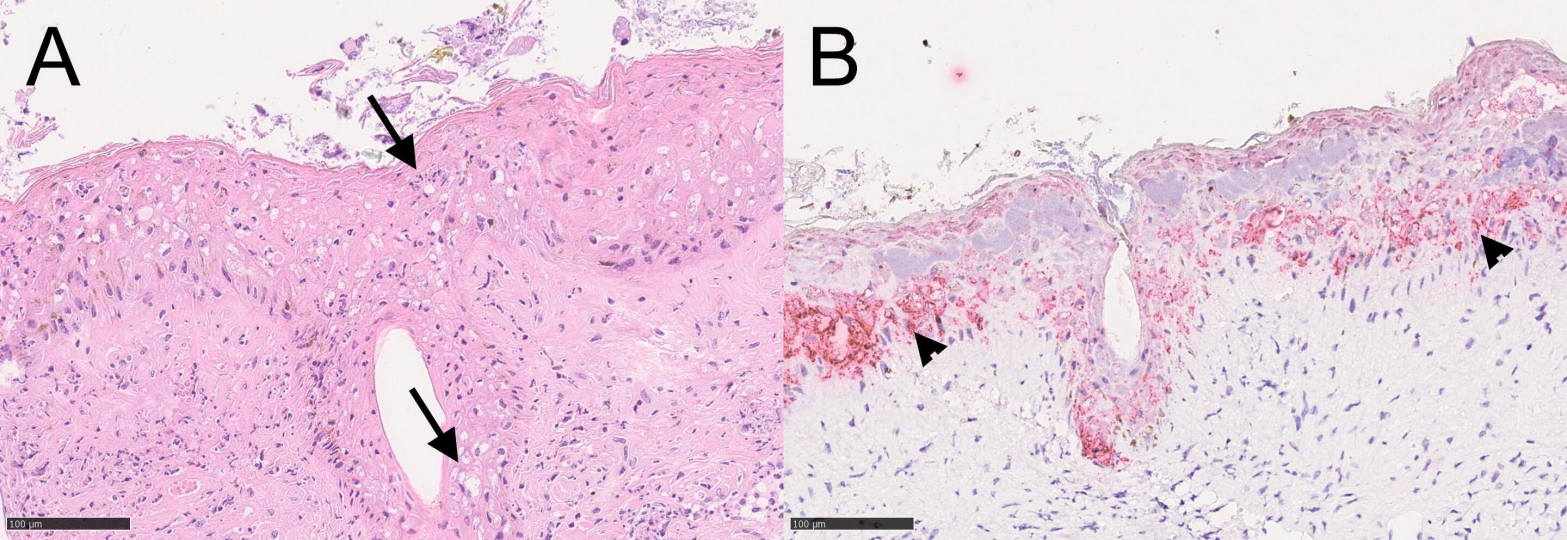
Microscopic images from eye lesions in semi-domesticated Eurasian tundra reindeer (*Rangifer t. tarandus*) infected with cervidpoxvirus. Microscopic images from eye lesion in reindeer No-22-80-P92. (**A**) Hematoxylin and eosin**-**stained section shows degenerated epithelium with vacuolization and necrosis in the epidermis (arrows). Microscopic image (**B**) from section stained with probe V-DPV-C1 using RNAscope *in situ* hybridization technique shows positive signals in the cytoplasm of epithelial cells of inflamed and degenerative epithelium, here seen as bright red stain (arrowheads). Bar 100 µm.

Histological sections of the eyelids of both reindeer showed a focally disrupted epidermis with the squamous epithelium lost or partly necrotic and covered with fibrin. There were several degenerate neutrophilic granulocytes, bacteria, and cell debris in the epidermis. Epithelial hyperplasia with cytoplasmic vacuolization and coagulative necrosis demarcated the line between healthy and disrupted epidermis. Some sections had intraepithelial pustules. There were multifocal to confluent areas with moderate to severe diffuse infiltration of inflammatory cells in the dermis, mainly neutrophilic granulocytes, but also lymphocytes and a few macrophages. This inflammation was profound and extended partly into the muscular layer. Hair follicles and sebaceous glands had degenerative to necrotic epithelium. In less affected areas, where the epidermis was still intact, there was a mild, mostly lymphocytic infiltrate, diffusely spread in the outer layer of the dermis. No inclusion bodies were identified (Fig. 4A and B).

### Transmission electron microscopy

**Fig 5:**
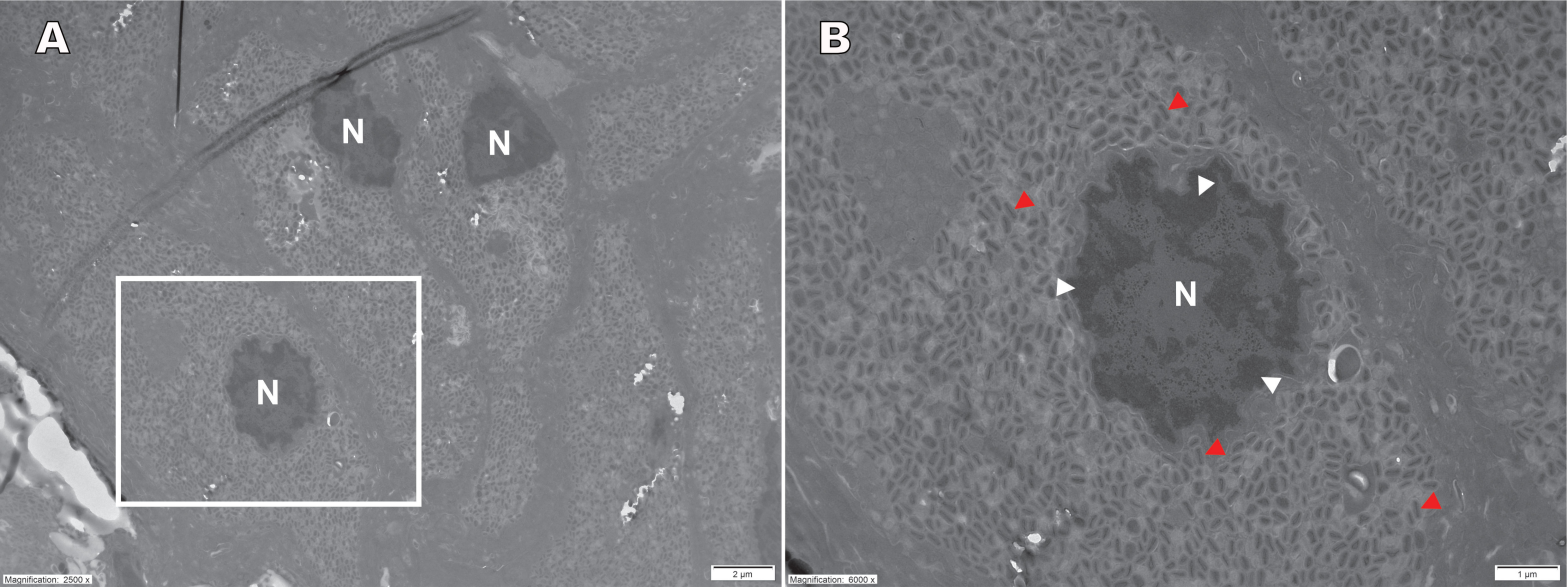
Transmission electron microscopy images from periorbital tissue from semi-domesticated Eurasian tundra reindeer (*Rangifer t. tarandus*) infected with cervidpoxvirus. Transmission electron micrographs (TEM) of the periorbital tissue from reindeer Sw 23-80-P207-16 showing epithelial cells with nuclear condensation (white arrow heads in the cell nucleus [N]) and intracytoplasmic localization of typical mature poxvirus particles with electron-dense, dumbbell-shaped cores and distinct outer envelopes (red arrowheads). Overview at 2,500× (**A**) and higher magnification at 6,000× (**B**).

TEM was performed on periorbital tissue samples from reindeer Sw-23-80-P207-16. The micrographs revealed epithelial cells exhibiting nuclear condensation, suggestive of cellular necrosis or apoptosis. Intracytoplasmic localization of mature poxvirus-like particles was observed (Fig. 5). These virions displayed the characteristic poxvirus morphology, with a membrane surrounding a biconcave core. Similar poxvirus-like particles were identified in tissue samples from the perianal lesions of reindeer No-22-80-P92. Similar to the results from reindeer Sw-23-80-P207-16, the virions were aggregated within the cytoplasm of infected epithelial cells. TEM analysis of cell pellets from cultures inoculated with homogenized crusts from both reindeer also demonstrated the presence of mature poxvirus-like particles (Fig. S2A and B).

### Shotgun sequencing pilot and identification of a cervidpoxvirus

Shotgun sequencing identified a virus closely related to DPV W-848-83 (GCF_000861985.1) in the *Cervidpoxvirus* genus (15). A partial draft genome was generated as described in Materials and Methods (results not shown). The sequencing depth for the partial draft genome was 5.7×.

We tentatively name the virus *Cervidpoxvirus reindeerpox* (CvRPV), following the binomial “genus-species” format approved by the International Committee on Taxonomy of Viruses (ICTV), but further characterization is needed to confirm whether this in fact is a new species.

### RNAscope *in situ* hybridization

RNAscope *in situ* hybridization analyses of eyelids from reindeer No-22-80-P92 and Sw-23-80-P207-16 revealed a strong cytoplasmic signal from the remaining degenerative epithelial cells in dermis and hair follicles (Fig. 4B).

### Polymerase chain reaction

The qPCR primers and probe were designed with a perfect match to the CvRPV partial draft genome, Moosepox virus GoldyGopher14 (GCA_006452215.1), White-tailed deer poxvirus OV179 (GCA_006451045.1), DPV W-1170-84 (GCA_006465565.1), and near perfect match (a single nucleotide polymorphism) with DPV W-848-83 (GCF_000861985.1). To allow for this potential mismatch, this position was degenerated in the reverse primer. To assess the specificity of the qPCR assay, the primers and probe were aligned to relevant poxvirus relatives of CvRPV (Fig. 1). The alignment showed 13–26 mismatches between the cervidpoxvirus qPCR assay and the related poxviruses. The amplicon was 137 base pairs long.

All tissues from reindeer No-22-80-P92 tested by qPCR were positive for the presence of cervidpoxvirus DNA, except for a retropharyngeal lymph node and the buffy coat sample. The virus was most abundant in skin lesions from the vulva (Cq 14.9) and eyelids (Cq 17.3, Table 1).

**TABLE 1:** qPCR results for cervidpoxvirus and estimation of relative virus amounts in various tissues from semi-domesticated Eurasian tundra reindeer (*Rangifer t. tarandus*) No-22-80-P92 infected with cervidpoxvirus

Tissue	Cq value	Linear ratio^*a*^ ,%
Eye crust	14.9	100
Vulva lesion	17.3	18.9
Eyelid lesion	19.5	4.1
Conjunctiva	23.1	0.34
Anal lesion	24.0	0.18
Prescapular lymph node (sample 1)	25.8	0.052
Uterus	26.3	0.037
Heart	27.0	0.023
Liver	29.2	0.005
Kidney	30.9	0.0015
Spleen	31.4	0.0011
Lung	31.4	0.0011
Prescapular lymph node (sample 2)	32.0	0.00071
Cerebellum	34.5	0.00013
Cerebrum	37.1	0.000021
Buffy coat	39.0	0.000006
Retropharyngeal lymph node	No Cq	NA^*b*^
Blood (5 samples)	No Cq	NA

^*a*^ The linear ratio was calculated with the ΔCq method (ratio = 100 × ((2^(Cq1) / (2^(Cq2))).

^*b*^ NA = Not assed, as virus was not detected.

Reindeer Sw-23-80-P207-16 had a Cq of 14.5 from a periorbital swab from lesions on the left eye (Fig. 4D). Two other reindeer from the same outbreak showed a Cq of 26.0 (Sw-23-80-P207-19) and 19.8 (Sw-23-80-P207-25), respectively, in periorbital swabs.

### *In vitro* infection of Madin-Darby bovine kidney cells

**Fig 6:**
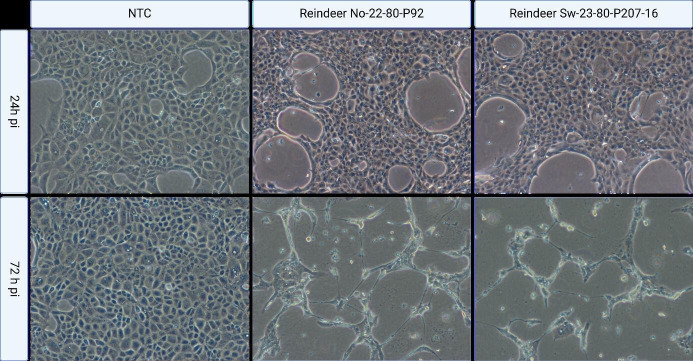
Cytopathic effects following *in vitro* inoculation with materials obtained from semi-domesticated Eurasian tundra reindeer (*Rangifer t. tarandus*) infected with cervidpoxvirus. Cytopathic effects (CPE) in Madin-Darby bovine kidney epithelial (MDBK) cells following inoculation with culture medium (non-target control, NTC, left column) or with materials obtained from sick reindeer—No-22-80-P92 (middle column)**,** Sw-23-–80-P207-16 (right column). Characteristic CPE included enlarged cytoplasm and cell rounding in the early time points, progressing to cell detachment and lysis, when nearly complete CPE was observed in inoculated wells. MDBK cell cultures were inoculated either with only culture medium (non-target control, NTC, left column) or with supernatant from the first passage of cell cultures previously inoculated with homogenized scabs from the perianal area of reindeer No-22-80-P92 (middle column) or from scabs from the eye lesions of reindeer Sw-23-80-P207-16 (right column). The images show cellular morphology and development of CPE (i.e., enlarged cytoplasm, cell rounding progressing to cell detachment and lysis). No CPE is visible in the NTC wells, while the inoculated wells show progressive CPE between 12 and 24 h p.i. and nearly complete CPE by 72 h p.i.

First passage: Madin-Darby bovine kidney (MDBK) cells showed various degrees of cytopathic effect (CPE) on day 3–5 post p.i. Characteristic CPE included enlarged cytoplasm and cell rounding in the early time points, progressing to cell detachment and lysis. The qPCR of the cell culture supernatant yielded Cq values of 22.9 and 23.1 for reindeer No-22-80-P92 and reindeer Sw-23-80-P207-16, respectively. Second passage: CPE was observed 24 h p.i. and increased in severity up to 72 h p.i., with almost 100% CPE at that time. Negative control cells did not present any visible damage (Fig. 6).

## DISCUSSION

This study reports the first detection of a virus from the genus *Cervidpoxvirus* in Europe, causing disease in semi-domesticated reindeer in Norway and Sweden.

The trimmed reads from semi-domesticated reindeer exhibiting periorbital lesions were mapped to the genome of the White-tailed deer poxvirus (MF966153.1) in a next-generation sequencing pilot experiment. A qPCR was designed specifically targeting the *Cervidpoxvirus* genus, employing primers that exhibited perfect alignment with the pilot draft genome and four published cervidpoxvirus genomes. Notably, at one position, the primers allowed for degeneracy, accepting either a C or T nucleotide. The qPCR amplification efficiency was 107.6% and demonstrated a linearity above 0.990, and the qPCR assay performance was, therefore, deemed acceptable. Brazilian porcupinepox virus 1 was the closest match, with 13 mismatches to primers and probe, making it unlikely to produce a false positive signal, and we concluded that the qPCR is specific for cervidpoxviruses. We observed variation in CvRPV loads across different tissues with Cq values ranging from 14.9 in eye lesions to 39.0 in buffy coat samples (Table 1), which exceeds the expected technical variability of PCR. All samples were collected fresh and handled consistently, minimizing procedural errors and ensuring high DNA quality.

The qPCR results show that samples from visible lesions on the animal exterior have higher viral loads. The eye crust from reindeer No-22-80-P92 had the lowest Cq value of 14.9, while the vulva lesion had a Cq value of 17.3. The eyelid lesion and conjunctiva showed Cq values of 19.5 and 23.1, respectively. These findings are consistent with previous studies in other poxvirus-infected ruminants, where samples from visible lesions typically show higher viral loads (42). It is interesting to note that the Cq values (14.5) of periorbital swab samples from one of the diseased animals were comparable to the Cq values from tissues from necropsied animals, such as eye crust (14.9), vulva lesion (17.3), and eyelid lesion (19.5) (Table 1), reflecting the amount of viral particles present, and indicating that swab samples from lesions of live animals may be of high value for diagnostic purposes.

Many of the clinical signs and pathological findings were in accordance with those previously described in reindeer in North America (17, 24), including varying degrees of periorbital ulcers, subsequent crust formation, and crusts around the genital area. Histopathological findings of the eyelids were also in accordance with previous descriptions (17). Moreover, the TEM findings confirm active replication of poxvirus in the affected tissues and support the diagnosis of a poxvirus infection in these reindeer. The presence of virions in both tissue samples, swab samples from mucosal membranes, and cultured cells indicated that the virus is capable of infecting and replicating within multiple cell types, contributing to the observed lesions.

It is, however, still unknown whether the virus previously causing disease in Toronto Zoo, Canada, and the virus causing disease in reindeer in Norway and Sweden are identical. Further characterization and comparison of the viruses is warranted, which may result in the cervidpoxvirus detected in Fennoscandian reindeer being proposed as a novel second virus species within the *Cervidpoxvirus* genus.

The enlarged lymph nodes, along with swollen liver and spleen, are indicative of a systemic immune reaction in the infected reindeer. The qPCR results showed that samples from internal organs such as the kidney, spleen, lung, cerebellum, and cerebrum had high Cq values (>30), suggesting low viral loads (Table 1), but still systemic viral presence. This suggests that the infection was generalized, which in poxviruses typically entails a phase of leukocyte-associated viremia, resulting in the spread of the virus to the skin and, to varying degrees, internal organs. Immunity to generalized poxvirus infections may be long-lasting (43). This corresponds to findings at Toronto Zoo, where the disease, after the initial outbreak, has occasionally reappeared in less severe forms, affecting immunologically naïve juvenile reindeer (24), indicating that immunity is developed after the first infection. This raises questions with regards to the herd immunity of Fennoscandian reindeer, which is currently unknown.

Cervidpoxviruses have previously been detected in white-tailed deer in Mississippi (25) and Florida, USA (26). In 1934 and 1948, nine white-tailed deer were imported from Minnesota, USA, to Finland and released into the wild. Today the population consists of approximately 100,000 animals in southern and central Finland (44, 45). The population does not geographically overlap with the semi-domesticated reindeer (5), but wild forest reindeer (*Rangifer tarandus fennicus*) are in contact with both white-tailed deer and semi-domesticated reindeer (46). It is theoretically possible that CvRPV was introduced to Finland with the white-tailed deer. There are no clinical signs of cervidpoxvirus infection reported from Finnish white-tailed deer or wild forest reindeer supporting disease occurrence in these populations. However, a serosurvey based on virus neutralization conducted in Oregon, USA, revealed a high prevalence (32%–59%) of cervidpoxvirus antibodies in apparently healthy black-tailed deer (*Odocoileus hemionus columbianus, n* = 55), mule deer (*Odocoileus hemionus hemionus, n* = 59), and Columbian white-tailed deer (*Odocoileus virginianus leucurus, n* = 50). This finding suggests that the virus may be more widespread in North American deer populations than previously thought and may only cause disease symptoms occasionally (47). The same could be the case for Finnish white-tailed deer and wild forest reindeer. Other sources of introduction to Fennoscandia, like through the import of roughage from the USA to Norway or Sweden, are also a theoretic possibility as poxviruses are highly stable in the environment (48).

When talking to reindeer herders, they recalled that similar periorbital lesions have occurred previously, however, only in single animals and on rare occasions. Whether these lesions were caused by a cervidpoxvirus remains unknown, but in other capreolinae, high seroprevalence has been detected in clinically healthy animals, indicating that the virus may circulate without causing disease (47). It may hence be the case that CvRPV was historically present and that the recent disease emergence is connected to environmental drivers like habitat disturbance (6) resulting in altered host-pathogen-vector interactions and altered viral ecosystem dynamics (11).

Sheeppox virus, goatpox virus (49), and lumpy skin disease virus (50) can be mechanically transmitted by insects. High concentrations of poxvirus in crust material, as shown for CvRPV, enable such transmission (42). Flies were a severe problem the summer prior to the cervidpoxvirus outbreak at Toronto Zoo (17) and before the outbreak in Norway in 2022 (pers. obs. H. Lian). As ectotherms, insects are highly sensitive to environmental changes (51). Research in mountainous areas in mid-Sweden has shown that climate-driven shifts in mountain arthropod communities have led to the migration of species to higher altitudes (52). Climatic changes leading to alterations in insect dynamics and distributions may play a role in the spread of CvRPV between reindeer.

Rodents are the natural host of many poxviruses (53). We have no information about the presence of CvRPV in rodents that are sympatric with reindeer (e.g., Norwegian lemming; *Lemmus lemmus*, and Northern red-backed vole, *Myodes rutilus*), or in ecosystems in general. It is, however, possible that rodents could play a role as virus reservoirs for CvRPV. Rodents were abundant prior to the outbreaks in reindeer both in Toronto Zoo and in the vicinity of the experimental animal facility in Norway.

Altered management of semi-domesticated reindeer, like increased feeding and prolonged fencing in winter due to reduced winter pasture availability, may also have caused increased disease transmission by increasing animal density, resulting in poor hygienic conditions (12). In addition, stress due to altered ecosystems and herding practices, and a potential allostatic overload, may play a role, resulting in challenged immunocompetence, increased susceptibility to, and shedding of, infectious agents, as well as increasing the severity of clinical signs and the risk for a poor prognosis (54).

Retrospective studies could reveal if and to what extent Fennoscandian reindeer have previously been exposed to CvRPV, or if there are indications of a more recent introduction. It is also relevant to address virus presence in other potential hosts or reservoirs, such as other Capreolinae and rodents. The role of insects in the disease dynamics also warrants further investigation. Furthermore, controlled experimental inoculation studies could reveal important characteristics of the biology of CvRPV infection in reindeer, such as incubation time, routes of spreading, healing, and supportive treatment. Additionally, the impact of allostatic overload and reindeer resilience on CvRPV dynamics should be explored.

## AFTER EPUB

*[This article was published on 22 May 2025 with errors in the reference citations. The citations were corrected in the current version, posted on 5 June 2025.]*

## SUPPLEMENTAL MATERIAL

Supplemental figures spectrum.02296-24-s0001.docx Fig. S1 and S2.

ASM does not own the copyrights to Supplemental Material that may be linked to, or accessed through, an article. The authors have granted ASM a non-exclusive, world-wide license to publish the Supplemental Material files. Please contact the corresponding author directly for reuse.
