# Supplementary material for: First detection of a cervidpoxvirus in Europe—disease occurrence in semi-domesticated Eurasian tundra reindeer (Rangifer tarandus tarandus) in Norway and Sweden
Source: Microbiol Spectr. 2025 May 22;13(7):e02296-24. doi: 10.1128/spectrum.02296-24 (PMC12210880; doi:10.1128/spectrum.02296-24)
Supplement: Supplemental figures — Fig. S1 and S2. [file spectrum.02296-24-s0001.docx]

**Supplementary Figure 1.** Regression analysis of a dilution curve for the cervidpoxvirus qPCR assay.


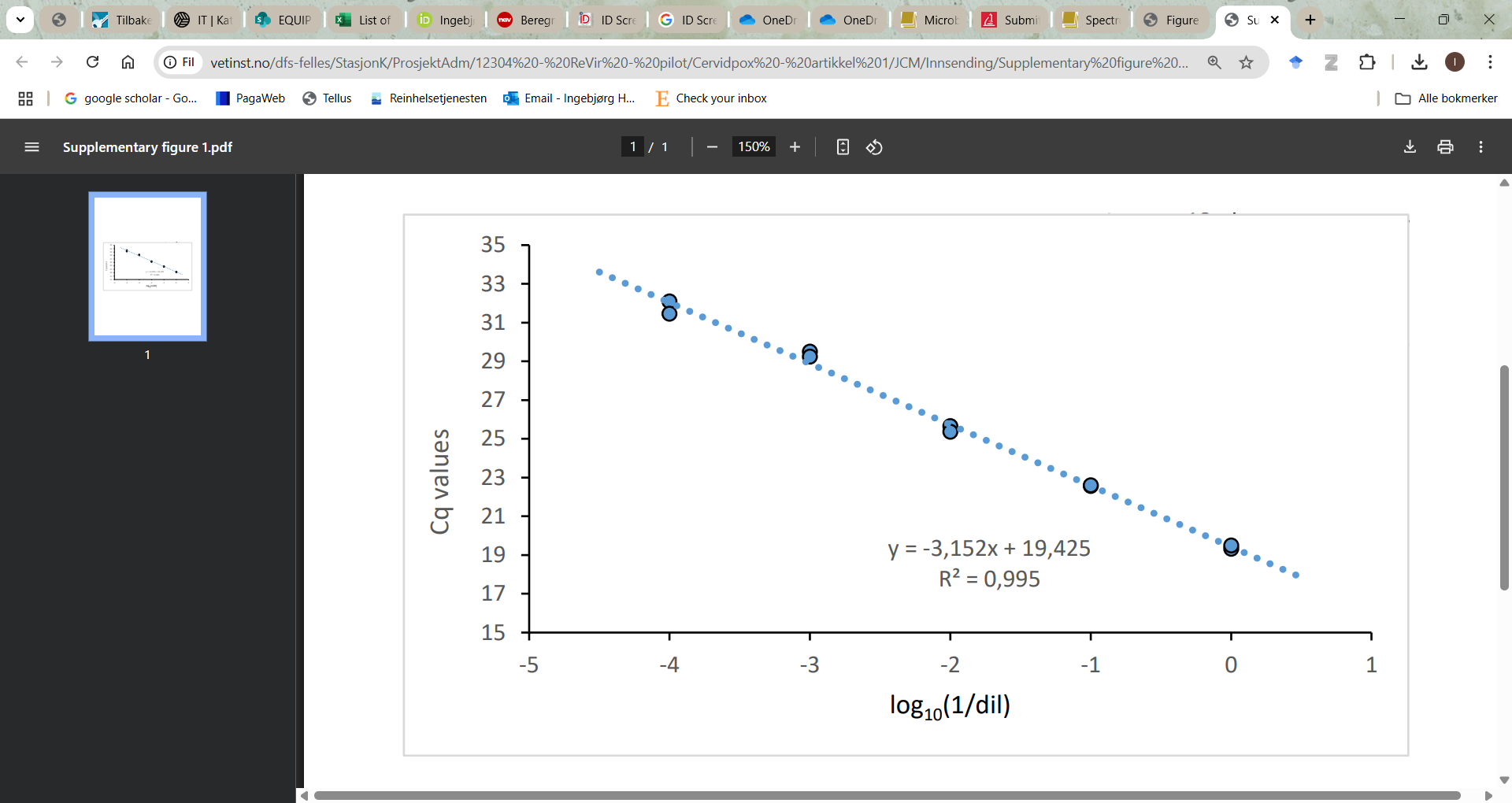


**Supplementary Figure 1.** Regression analysis of a dilution curve for the cervidpoxvirus qPCR assay. Linear regression gave an equation: y = -3.152x + 19.42 with linearity R² = 0.995

**Supplementary figure 2.** Transmission electron microscopy (TEM) images of cell debris pellets.


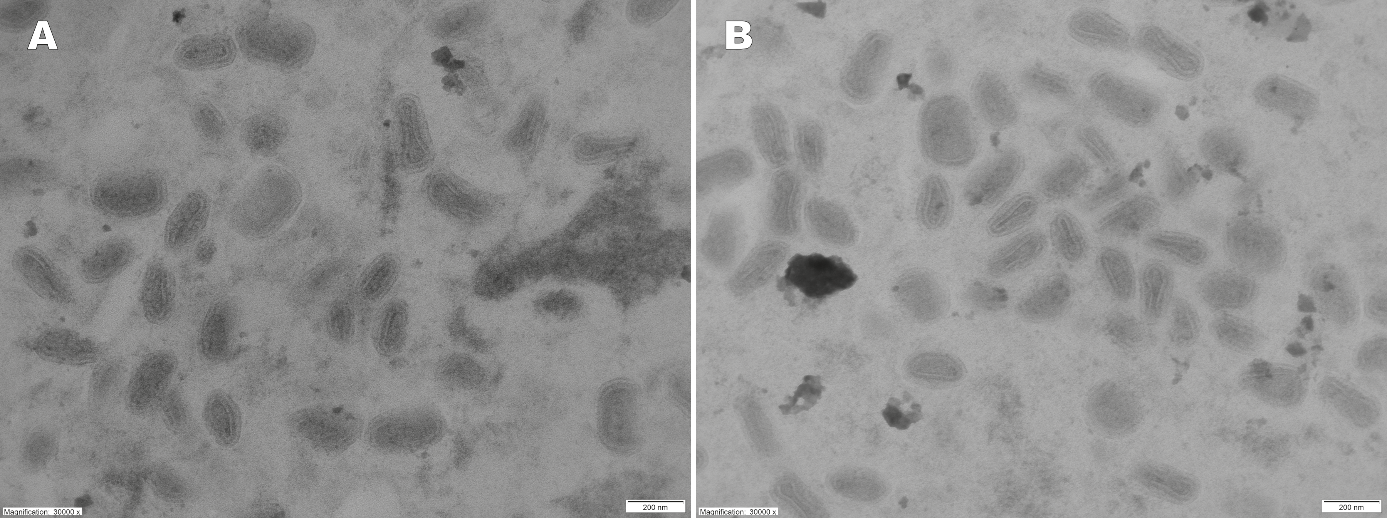
**Supplementary figure 2.** Transmission electron microscopy (TEM) images of cell debris pellets 3 days post inoculation from cell cultures inoculated with homogenized crusts from the periorbital lesions of reindeer Sw-23-80-P207-16 (A) and the perianal lesions of reindeer No-22-80-P92 (B) showing characteristic mature poxvirus particles with electron-dense, dumbbell-shaped cores and distinct outer envelopes.
